# Anti-Inflammatory Effects of* Aurantiochytrium limacinum* 4W-1b Ethanol Extract on Murine Macrophage RAW264 Cells

**DOI:** 10.1155/2019/3104057

**Published:** 2019-01-28

**Authors:** Shinya Takahashi, Masaki Yoshida, Makoto M. Watanabe, Hiroko Isoda

**Affiliations:** ^1^Faculty of Life and Environmental Sciences, University of Tsukuba, Tsukuba City 305-8572, Japan; ^2^Master's/Doctoral Program in Life Science Innovation, School of Integrative and Global Majors (SIGMA), University of Tsukuba, Tsukuba City 305-8572, Japan; ^3^Alliance for Research on the Mediterranean and North Africa (ARENA), University of Tsukuba, Tsukuba City 305-8572, Japan; ^4^Algae Biomass and Energy System R&D Center (ABES), University of Tsukuba, Tsukuba City 305-8572, Japan

## Abstract

*Aurantiochytrium limacinum* 4W-1b (AL4W-1b) is a newly discovered microalgal strain with unique features. In the present study, we investigated the effects of ethanol extracts of AL4W-1b on lipopolysaccharide- (LPS-) induced inflammatory responses in RAW264 murine macrophage cells. Pretreatment of RAW264 cells with the AL4W-1b extract significantly reduced the production of LPS-induced nitric oxide (NO) and the expression of proinflammatory cytokine genes, including tumor necrosis factor *α*, interleukin- (IL-) 1*β*, and IL-6. Treatment with the AL4W-1b extract also decreased the production of IL-1*β* and IL-6. These results suggest that AL4W-1b might have anti-inflammatory effects in RAW264 cells. The NF-*κ*B inhibitor, BAY 11-7082, synergistically prevented LPS-induced NO production after pretreatment with the AL4W-1b extract. Thus, the AL4W-1b extract may affect not only the NF-*κ*B pathway but also other inflammatory pathways. To the best of our knowledge, this is the first study to report the anti-inflammatory effects of AL4W-1b extract and its mechanism of action in LPS-stimulated murine macrophage cells.

## 1. Introduction

Inflammatory response is an important mechanism for host defense, but chronic inflammation is the underlying cause of several diseases including atherosclerosis, dementia, and cancer. Macrophages are the primary proinflammatory cells, and during inflammation, they produce inflammatory mediators such as nitric oxide (NO) and inflammatory cytokines. Although nonsteroidal anti-inflammatory drugs (NSAIDs) are generally used for chronic inflammation, they have undesirable side effects when chronically used. Therefore, there is a critical need to identify natural products with anti-inflammatory properties that can be used as a substitute.

In recent years, microalgae have been attracting attention not only as new biomass energy but also as a health food and novel medicine. A water-extracted fraction of* Botryococcus braunii* showed biological activities of dermatological interest [1]. Additionally, an ethanol extract of* B. braunii* showed antidepressant-like effects in a mouse behavior test [2]. Several studies have reported the anti-inflammatory effects of microalgal extracts on mammalian cells [3–5]. However, it is still necessary to explore more potent and biologically active novel compounds from these organisms.

Heterotrophic microalga* Aurantiochytrium *belongs to the thraustochytrid family and has been reported to contain an abundance of bioactive substances [6, 7]. The* Aurantiochytrium mangrovei* 18W-13a (AM18W-13a) strain has very high efficiency of hydrocarbon (e.g., squalene) production [7]. In a previous study, we showed the anti-inflammatory effects of microalgal strain AM18W-13a on murine macrophage RAW264 cells [8].

To identify novel microalgae possessing anti-inflammatory effects, we evaluated the anti-inflammatory effects of microalgal species other than AM18W-13a using our system. In this study, we examined the effects of algal ethanol extracts from the following microalgae:* B. braunii*,* Parietichytrium sarkarianum*,* Euglena gracilis,* and* Aurantiochytrium limacinum* 4W-1b (AL4W-1b).* B. braunii* BOT-22 is an autotrophic microalga that is known to produce extracellular hydrocarbons [9].* E. gracilis* is also an autotrophic microalga that has been shown to accumulate polysaccharides such as *β*-1,3-glucan (paramylon), which acts as an immune-enhancer [10, 11].* P. sarkarianum*, a type of thraustochytrid, accumulates long chain n-3 polyunsaturated fatty acids (PUFA); however, it seems that its accumulation of docosahexaenoic acids (DHA, 22:6n-3), a type of PUFA, is lower than that by* Aurantiochytrium* [12, 13]. AL4W-1b produces lipids and fatty acids, including the DHA, docosapentaenoic acid (DPA, 22:5n-3), and palmitic acid (16:0), but it shows lower production of squalene [14]. DHA has various physiological functions, such as anti-inflammatory, antidiabetic, and antidepression effects, and has been commercially used as a dietary supplement. DPA is also a kind of PUFA and has inhibitory effects on angiogenesis and platelet aggregation [15, 16]. The main source of DHA is fish oil, and other sources of DPA include seal meat and salmon, but thraustochytrids might also be a source of these PUFAs [17, 18]. To investigate the effects of functional compounds on a proinflammatory model system, lipopolysaccharide- (LPS-) stimulated RAW264 cells were employed [8]. Among the four types of microalgal extracts, we found that the AL4W-1b extract had the most effective anti-inflammatory activity. In the present study, we evaluated, for the first time, the anti-inflammatory activity of ethanol extracts of the AL4W-1b strain in LPS-stimulated murine macrophage RAW264 cells.

## 2. Materials and Methods

### 2.1. Preparation of Microalgal Extracts

All samples of microalgae, AL4W-1b,* B. braunii* BOT-22,* P. sarkarianum* 6F-10b,* E. gracilis* EOD-1, and AM18W-13a (provided by the Algae Biomass and Energy System R&D Center, University of Tsukuba, Japan) were lyophilized. The lyophilized powdered algal (0.5 g) extracts were obtained by adding 5 mL of 99.5% ethanol (EtOH) to the lyophilized powder and keeping the solution in a dark at room temperature for two weeks. After centrifugation, the supernatant of each extracted sample was filtered using a 0.22 *μ*m filter unit. The final solution of the extract was then stored in the dark at -80°C until use.

### 2.2. Cell Culture of RAW264 Cells

RAW264 murine macrophage cells (RCB0535, RIKEN BRC, Tsukuba, Japan) were cultured in Dulbecco's modified Eagle's Medium (DMEM) supplemented with 10% heat-inactivated fetal bovine serum (FBS) and 1% penicillin-streptomycin at 37°C in a humidified incubator containing 5% CO_2_. Cells were seeded in 96-well cell culture plates at a density of 2.0 × 10^4^ cells per well and were incubated at 37°C for 24 h.

### 2.3. Cell Proliferation Assay

The effects of the microalgal extracts on cell proliferation were determined by the mitochondrion-dependent reduction of 3-(4,5-dimethylthiazol-2-yl)-2,5-diphenyl tetrazolium bromide (MTT) to formazan [19] as described in our previous study [8]. In brief, RAW264 cells were treated with or without AL4W-1b extract at concentrations ranging from 1/10,000 to 1/100 at 37°C for 24 h. After the treatment, MTT solution was added to each well and incubated at 37°C for 4 h. After dissolving the resulting formazan crystals in each sample using 5% SDS, the absorbance was measured at 570 nm using a microplate reader (Power Scan HT, BioTEK Japan Inc.). The values were calculated as a percentage (%) of the control.

### 2.4. Measurement of NO Production

The concentration of nitrite oxide (NO) was measured using a Griess diazotization reaction [20] as described in our previous study [8]. In brief, RAW264 cells were treated with or without microalgal extract at 37°C for 24 h. After the pretreatment, LPS solution (1 ng/mL) was added to RAW264 cells and cells were incubated at 37°C for 12 h. Then, the supernatants of cell culture medium were used in a Griess reaction assay. The absorbance was measured at 540 nm using a microplate reader.

### 2.5. Gene Expression and Protein Expression Analysis

Gene expression and protein analysis was performed as described by Takahashi et al. [8]. For the quantification of mRNA, TaqMan real-time polymerase chain reaction (PCR) amplification reactions were performed using an Applied Biosystems 7500 Fast Real-Time System (Thermo Fisher Scientific Inc., Waltham, MA). All primers and the TaqMan Universal PCR Master Mix were obtained from Thermo Fisher Scientific. Specific primers for the mouse genes* Gapdh *(Mm99999915_g1),* interleukin-* (*IL-) 6* (Mm00446190_m1),* TNFα* (Mm00443258_m1), and* IL-1β* (Mm00434228_m1) were used. Gene expression levels were normalized to the* Gapdh* expression level.

To measure the protein expression, the amounts of IL-6 and IL-1*β* in RAW264 cells were assayed in the supernatant of the cell culture medium using the Bio-Plex Pro™ Mouse Cytokine Assay kit (BIO-RAD, USA) with a MAGPIX xPONENT 4.2 system (Merck Millipore Co, USA).

### 2.6. Treatment with the NF-*κ*B Inhibitor

RAW264 cells were pretreated with AL4W-1b extract for 24 h and treated with or without BAY 11-7082 (WAKO Pure Chemical), an NF-*κ*B inhibitor, at 10 *μ*M before treatment with LPS. After 12 h of treatment with LPS, NO production was measured.

### 2.7. Statistical Analysis

Statistical evaluations were performed using Student's* t-*test. Values were considered statistically significant if* p* < 0.01.

## 3. Results and Discussion

**Figure 1:**
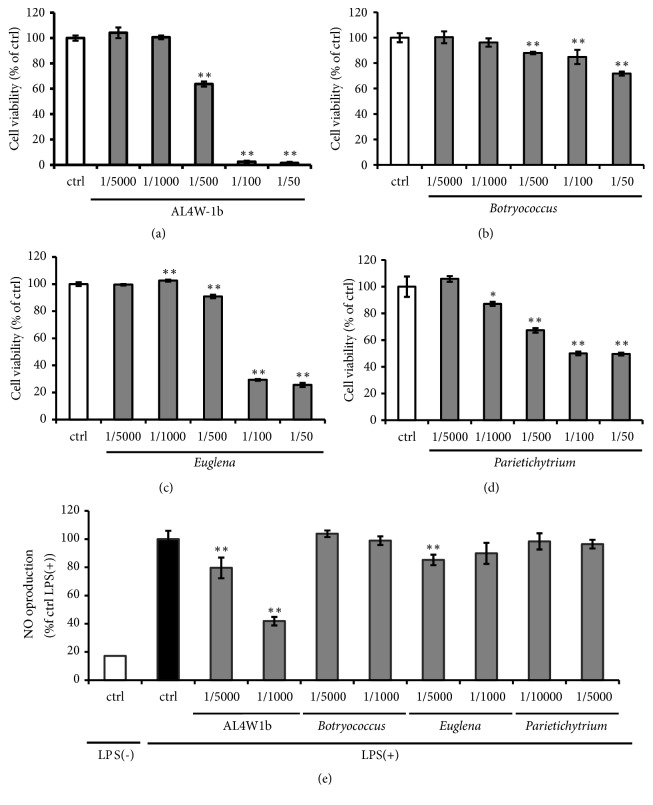
Evaluation of cytotoxicity on anti-inflammatory effects and nitrite oxide production in mouse RAW 264 cells pretreated with several algal ethanol extracts. RAW264 cells were treated with algal ethanol extracts at concentrations of 1/5000, 1/1000, 1/500, 1/100, and 1/50 of* Aurantiochytrium* 4W-1b (a),* Botryococcus braunii* BOT-22 (b),* Euglena gracilis* EOD-1 (c), and* Parietichytrium sarkarianum* 6b-10F (d) for 24 h. After treatment, cell proliferation was measured by MTT assay. Values are expressed as the mean ± SD for triplicate experiments and are expressed as a percentage of the control. (e) Cells were treated with algal ethanol extracts at concentrations of 1/5000 or 1/1000 for 24 h. After treatment, cells were activated with LPS (1 ng/mL) for 12 h. The amount of NO production was measured by Griess reaction. Values are expressed as the mean ± SD of triplicate experiments and are expressed as a percentage of the control LPS (+). The asterisk indicates a mean value that is significantly different from that of the control group (*∗∗ p *< 0.01).

First, we checked the cell proliferation and anti-inflammatory abilities of ethanol extracts of four species of microalgae including AL4W-1b. The cytotoxicities of the ethanol extracts of four species of microalgae were evaluated by MTT assay. The ethanol extracts of the algal samples were diluted at 0.0002, 0.001, 0.002, 0.01 and 0.02% (1/5000, 1/1000, 1/500, 1/100, and 1/50, respectively) in culture medium. In all algal samples, concentrations ranging from 1/5000 to 1/1000 did not affect the viability of the RAW264 cells (Figures 1(a)–1(d)). Therefore, algal samples diluted at 1/5000 and 1/1000 were used for the following assays.

We measured NO production of the LPS-stimulated RAW264 cells pretreated with the algae samples. Only pretreatment with 1/5000 and 1/1000 of AL4W-1b extract caused a reduction, by about 80% and 40%, respectively, in NO production as compared with no pretreatment of the AL4W-1b extract (Figure 1(e)). Therefore, the AL4W-1b extract had positive effects on anti-inflammatory effects with regard to LPS-stimulation-related schemes.

**Figure 2:**
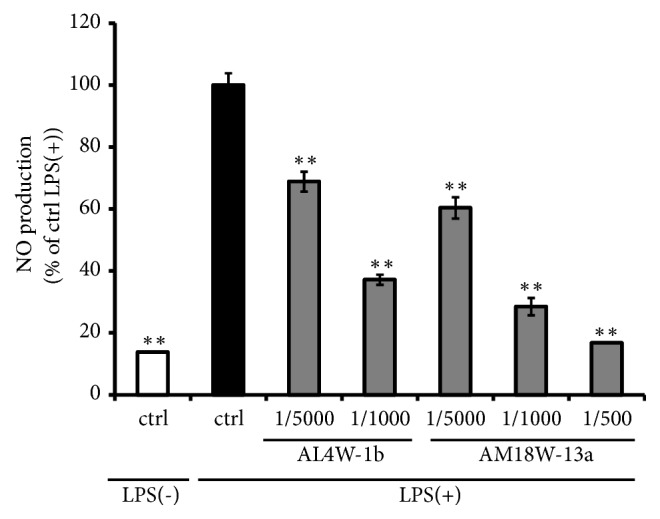
Comparison of reduction in nitrite oxide production in mouse RAW 264 cells pretreated with ethanol extracts of AL4W-1b and AM18W-13a. RAW264 cells were pretreated with ethanol extracts of AL4W-1b and AM18W-13a at concentrations of 1/5000 and 1/1000 for 24 h. After pretreatment, the cells were activated with LPS (1 ng/mL) for 12 h. NO production was measured by Griess reaction. Values are expressed as the mean ± SD of triplicate experiments and are expressed as a percentage of the control LPS (+) treatment. The asterisk indicates a mean value that is significantly different from that of the control group (*∗∗ p *< 0.01).

In addition, we compared the effects of ethanol extract originating from AL4W-1b and AM18W-13a on LPS-stimulated NO production in RAW264 cells. The AL4W-1b decreased NO production but was slightly less effective than AM18W-13a (Figure 2).

**Figure 3:**
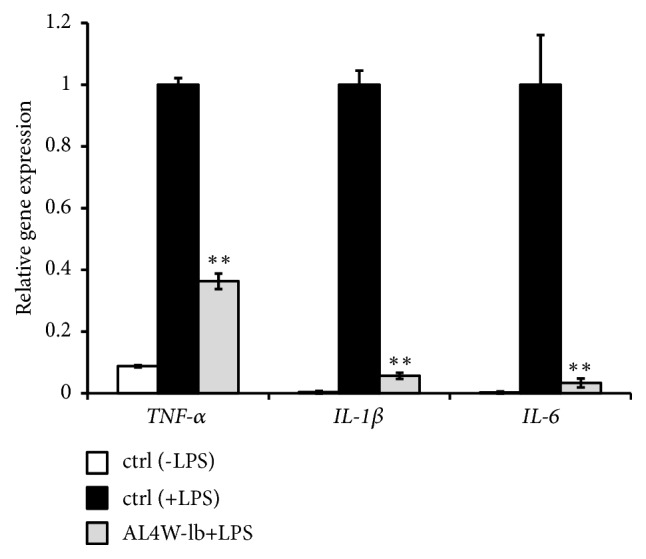
Effects of pretreatment with AL4W-1b extract on gene expression of* TNF-α, IL-1β, *and* IL-6 *in RAW264 cells. RAW264 cells were pretreated with or without 1/1000 AL4W-1b for 24 h. After pretreatment, the cells were activated with LPS (1 ng/mL) for 3 h. After treatment, the gene expression levels of* TNF-α, IL-1β, and IL-6 *were evaluated using RT-PCR. Values are expressed as the mean ± SD of triplicate experiments. The asterisks indicate a mean value that is significantly different from that of the control group (*∗∗ p *< 0.01).

To determine the effects of the AL4W-1b extract on the expression of proinflammation cytokines, we measured the gene expression of* TNF-α*,* IL-1β,* and* IL-6* in LPS-stimulated cells with the AL4W-1b extract pretreatment by the real-time reverse transcription (RT) PCR method. Pretreatment with 1/1000 AL4W-1b extract suppressed expression of these genes as compared to those in only LPS-stimulated cells (Figure 3).

**Figure 4:**
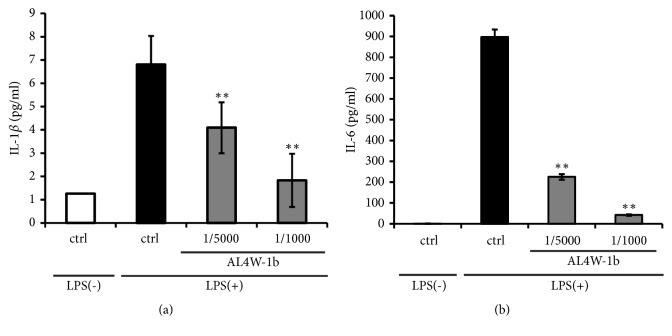
Effects of pretreatment with AL4W-1b extract on proinflammatory cytokines in RAW264 cells. Amounts of (a) IL-1*β* and (b) IL-6 production in RAW264 cells are shown. Cells were pretreated with or without AL4W-1b extract at concentrations of 1/5000 and 1/1000 for 24 h. After treatment, cells were activated with LPS (1 ng/mL) for 12 h. The secreted proinflammatory mediators were determined by ELISA assay with the MAGPIX xPONENT system. Values are expressed as the mean ± SD of duplicate experiments. A mean value that is significantly different from that of the control LPS (+) group is indicated as *∗∗ p *< 0.01.

We also measured amounts of two proinflammatory cytokine proteins, IL1*β* and IL6, in the AL4W-1b extract pretreated cells by the ELISA method. Pretreatments with 1/5000 and 1/1000 AL4W-1b extract reduced expression of IL1*β* by 60.2% and 26.9%, respectively, compared to those cells stimulated with LPS alone (Figure 4(a)). Pretreatments with 1/5000 and 1/1000 AL4W-1b extract also reduced the expression of IL-6 by 25.1% and 4.7%, respectively (Figure 4(b)). These results show that AL4W-1b extract can suppress proinflammatory related genes and the amounts of their translated proteins.

In this study, we showed that AL4W-1b extract suppressed NO production and protein and gene expression of proinflammatory cytokines. According to these results, the AL4W-1b extract also possesses anti-inflammatory properties.

G protein-coupled receptor 120 (GPR120) is known as a receptor of omega-3 fatty acids, including DHA, and mediates anti-inflammatory effects in RAW264.7 cells [21]. In recent reports, DHA was shown to activate cytosolic phospholipase A2 (cPLA2), cyclooxygenase 2 (COX2), and prostaglandin E2 (PGE2) production via GPR120, and resulted in the inhibition of IL-6 production via the NF-*κ*B pathway, which is an important proinflammatory pathway for mammalian cells, in RAW264.7 cells [22]. DPA also suppresses the gene expression of* IL-6*,* IL-1β*,* iNOS*, and* cox-2*, which are known to be proinflammatory mediators, in LPS-stimulated RAW264.7 cells [18]. On the other hand, palmitic acid activates proinflammatory signaling, which is triggered by Toll-like receptor (TLR) 4 and TLR2 in RAW264.7 cells [23].

In a previous study, we showed that an ethanol extract of the AM18W-13a strain had anti-inflammatory effects on RAW 264 cells [8]. The AM18W-13a strain is rich in hydrocarbons and squalene, whereas it has a relatively low abundance of lipid compounds [7, 24]. AL4W-1b is rich in the fatty acids DHA, DPA, and palmitic acid, which constitute 17.2–27.9%, 3.6–3.9%, and 46.9–52.6% of total fatty acids, respectively, and the concentrations of which vary depending on glucose concentration in the growth medium [14]. These previous reports indicate that AL4W-1b contains DHA and DPA as the major PUFA components [14]. Thus, we predicted that the DHA and/or DPA accumulated in AL4W-1b would show anti-inflammatory effects on LPS-stimulated proinflammation in RAW264 cells. The AL4W-1b showed anti-inflammatory effects but was slightly less effective than AM18W-13a. AM18W-13a also accumulates DHA and DPA [25]. AM18W-13a is rich in squalene and has fewer triglycerides, while AL4W-1b is abundant in triglycerides but has very low amounts of squalene [7]. Thus, we predicted that the squalene and some types of triglycerides were active compounds in the AM18W-13a and AL4W-1b strains, respectively. These two strains may possess different active compounds with regard to their respective inflammatory effects. However, we have not clarified the actual active compounds of either AL4W-1b or AM18W-13a. Further research is needed to identify the active compounds in these two strains.

**Figure 5:**
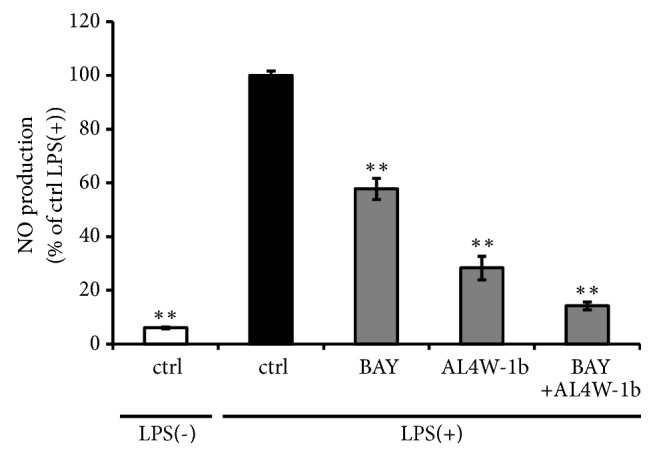
Reduction in nitrite oxide production in mouse RAW 264 cells pretreated with an ethanol extract of AL4W-1b and an inhibitor of the NF-*κ*B pathway, BAY 11-7082. RAW264 cells were pretreated with AL4W-1b extracts at a concentration of 1/1000 for 24 h. After treatment, the cells were treated with or without 10 *μ*M BAY11-7082 (BAY) for 30 min, and then cells were activated with LPS (1 ng/mL) for 12 h. The amount of NO produced was measured by the Griess reaction. Values are expressed as the mean ± SD of triplicate experiments and are expressed as a percentage of the control LPS (+) treatment. The asterisks indicate a mean value that is significantly different from that of the control group (*∗∗ p *< 0.01).

In this study, we did not clarify the mechanisms of the reduction in proinflammatory response of AL4W-1b extracts. In preliminary experiments, we observed that treatment with BAY11-7082, which is known to inhibit the dephosphorylation of I*κ*B and translocation of NF-*κ*B [26], synergistically prevented the proinflammatory responses with AL4W-1b extract. We found that pretreatment of RAW264 cells with BAY11-7082 after treatment with the AL4W-1b extracts more effectively suppressed LPS-stimulated NO production compared to pretreatment with BAY11-7082 or AL4W-1b extracts alone (Figure 5). Based on this result, the AL4W-1b extract may inhibit not only the NF-*κ*B pathway but also other pathways concerned with inflammatory effects; e.g., the MAPK pathway or PI3K pathways. In the future, we need to clarify the evidence regarding these.

## 4. Conclusions

In conclusion, we evaluated nitrite oxide (NO) production and proinflammatory cytokine expression in algal extract-treated cells. Ethanol extracts of the AL4W-1b cells showed suppression of LPS-induced NO production and expression of proinflammatory cytokines. These results suggest that AL4W-1b extracts possess anti-inflammatory properties.

## Data Availability

The data used to support the findings of this study are available from the corresponding author upon request.
